# Data‐Driven Implementation Strategy to Optimise Clinician Behaviour Change at Scale in Complex Clinical Environments: A Multicentre Emergency Care Study

**DOI:** 10.1111/jan.16461

**Published:** 2024-09-15

**Authors:** Kate Curtis, Belinda Kennedy, Julie Considine, Margaret Murphy, Sarah Kourouche, Mary K. Lam, Ramon Z. Shaban, Christina Aggar, James A. Hughes, Margaret Fry

**Affiliations:** ^1^ Faculty of Medicine and Health The University of Sydney Susan Wakil School of Nursing and Midwifery Camperdown New South Wales Australia; ^2^ Emergency Services, Illawarra Shoalhaven Local Health District Wollongong Hospital Wollongong New South Wales Australia; ^3^ School of Nursing and Midwifery and Centre for Quality and Patient Safety Research in the Institute for Health Transformation Deakin University Geelong Victoria Australia; ^4^ Centre for Quality and Patient Safety Research—Eastern Health Partnership Box Hill Victoria Australia; ^5^ Western Sydney Local Health District North Parramatta New South Wales Australia; ^6^ School of Health and Biomedical Sciences RMIT University Melbourne Victoria Australia; ^7^ Sydney Institute for Infectious Diseases, Faculty of Medicine and Health The University of Sydney Camperdown New South Wales Australia; ^8^ Research and Education Network & District Executive Western Sydney Local Health District North Parramatta New South Wales Australia; ^9^ New South Wales Biocontainment Centre Western Sydney Local Health District and New South Wales Health North Parramatta New South Wales Australia; ^10^ Northern NSW Local Health District Lismore New South Wales Australia; ^11^ Faculty of Health Southern Cross University Bilinga Queensland Australia; ^12^ School of Nursing, Centre for Healthcare Transformation Queensland University of Technology Bilinga Queensland Australia; ^13^ University of Technology Sydney Faculty of Health Sydney New South Wales Australia; ^14^ Northern Sydney Local Health District St Leonards New South Wales Australia

**Keywords:** analgesia, clinical deterioration, emergency nursing, emergency service, hospital, mixed‐methods, nursing assessment, patient assessment, patient outcome assessment, randomised controlled trial

## Abstract

**Aim:**

To develop an evidence‐driven, behaviour change focused strategy to maximise implementation and uptake of HIRAID (History including Infection risk, Red flags, Assessment, Interventions, Diagnostics, communication and reassessment) in 30 Australian rural, regional and metropolitan emergency departments.

**Design:**

An embedded, mixed‐methods study.

**Methods:**

This study is the first phase of a step‐wedge cluster randomised control trial of HIRAID involving over 1300 emergency nurses. Concurrent quantitative and qualitative data were collected via an electronic survey sent to all nurses to identify preliminary barriers and enablers to HIRAID implementation. The survey was informed by the Theoretical Domains Framework, which is a synthesis of behavioural change theories that applies the science of intervention implementation in health care to effect change. Quantitative data were analysed using descriptive statistics and qualitative data with inductive content analysis. Data were then integrated to generate barriers and enablers to HIRAID implementation which were mapped to the Theoretical Domains Framework. Corresponding intervention functions and Behaviour Change techniques were selected and an overarching implementation strategy was developed through stakeholder consultation and application of the APEASE criteria (Affordability, Practicability, Effectiveness and cost‐effectiveness, Acceptability, Side‐effects/safety and Equity).

**Results:**

Six barriers to HIRAID implementation were identified by 670 respondents (response rate ~58%) representing all 30 sites: (i) lack of knowledge about HIRAID; (ii) high workload, (iii) lack of belief anything would change; (iv) not suitable for workplace; (v), uncertainty about what to do and (vi) lack of support or time for education. The three enablers were as follows: (i) willingness to learn and adopt something new; (ii) recognition of the need for something new and (iii) wanting to do what is best for patient care. The 10 corresponding domains were mapped to seven intervention functions, 21 behaviour change techniques and 45 mechanisms. The major components of the implementation strategy were a scaffolded education programme, clinical support and environmental modifications.

**Conclusions:**

A systematic process guided by the behaviour change wheel resulted in the generation of a multifaceted implementation strategy to implement HIRAID across rural, regional and metropolitan emergency departments. Implementation fidelity, reach and impact now require evaluation.

**Impact:**

- HIRAID emergency nursing assessment framework reduced clinical deterioration relating to emergency care and improved self‐confidence and documentation in emergency departments in pilot studies. Successful implementation of any intervention in the emergency department is notoriously difficult due to workload unpredictability, the undifferentiated nature of patients and high staff turnover.
- Key barriers and enablers were identified, and a successful implementation strategy was developed.
- This study uses theoretical frameworks to identify barriers and enablers to develop a data‐driven, behavioural‐focused implementation strategy to optimise the uptake of HIRAID in geographically diverse emergency departments which can be used to inform future implementation efforts involving emergency nurses.

**Reporting Method:**

The CROSS reporting method (Supporting Information S3) was used to adhere to EQUATOR guidelines.

**Patient or Public Contribution:**

No Patient or Public Contribution.

**Trial Registration:**

Australian New Zealand; Clinical Trials Registry (ANZCTR) number: ACTRN12621001456842, registered 25/10/2021

**Abbreviations**
APEASE criteria: Affordability, Practicability, Effectiveness and cost‐effectiveness, Acceptability, Side‐effects/safety and Equity
BCTT: Behaviour Change Techniques Taxonomy
CNUMS: Clinical Managers
HIRAID: History including Infection risk, Red flags, Assessment, Interventions, Diagnostics
NUM: Nurse Unit Managers
REDCap: Research Electronic Data Capture

## 1 Background

Demand for emergency care is increasing globally (Curtis et al. 2020). The emergency care environment is uniquely challenging with unpredictable workloads, undifferentiated patients and overcrowding (Fry et al. 2023). Emergency nurses are the first emergency department clinicians to assess patients and their practice is fundamental to patient safety and quality. Emergency nurses are responsible for the initial and ongoing assessment and management of patients of all ages, with varying degrees of clinical urgency and illness or injury severity. Patient safety is contingent on the emergency nurses' accurate assessment, interpretation of clinical data, intervention and escalation of care for deteriorating patients (Fasoli 2010).

Internationally, there is increasing emergency demand resulting in significant delays to patient care (Razzak, Usmani, and Bhutta 2019; Morley et al. 2018). In 2021–2022 there were around 8.79 million presentations to Australia's 292 emergency departments (Australian Institute of Health and Welfare [AIHW] 2023). Many of these patients experienced significant delays in assessment and management. National emergency department performance reports consistently show that less than two‐thirds of urgent (Australasian Triage Scale category 3) patients are seen by a medical officer within the 30‐min recommended timeframe (AIHW 2023; Australian Institute of Health and Welfare 2021). While emergency department patients are waiting for medical assessment, they are solely in the care of emergency nurses. Further, emergency nurses are also responsible for ongoing patient assessment and care, and escalation of care for deteriorating patients after a patient has been assessed by a medical officer.

Nonetheless, Australian emergency nurses' approach to patient assessment is inconsistent, and there is significant unwarranted variation in nursing documentation, communication and care (Jones, Shaban, and Creedy 2015). There are reports of avoidable patient deterioration, poor pain management and patient dissatisfaction with emergency care (Considine et al. 2017; Forster et al. 2007; Buchan et al. 2016). To address these problems, the HIRAID emergency nursing framework was designed (Munroe et al. 2015; Curtis et al. 2009) and pilot‐tested in the simulated environment (Munroe et al. 2016) and four emergency departments in New South Wales, Australia (Curtis, Munroe, et al. 2021).

**FIGURE 1:**
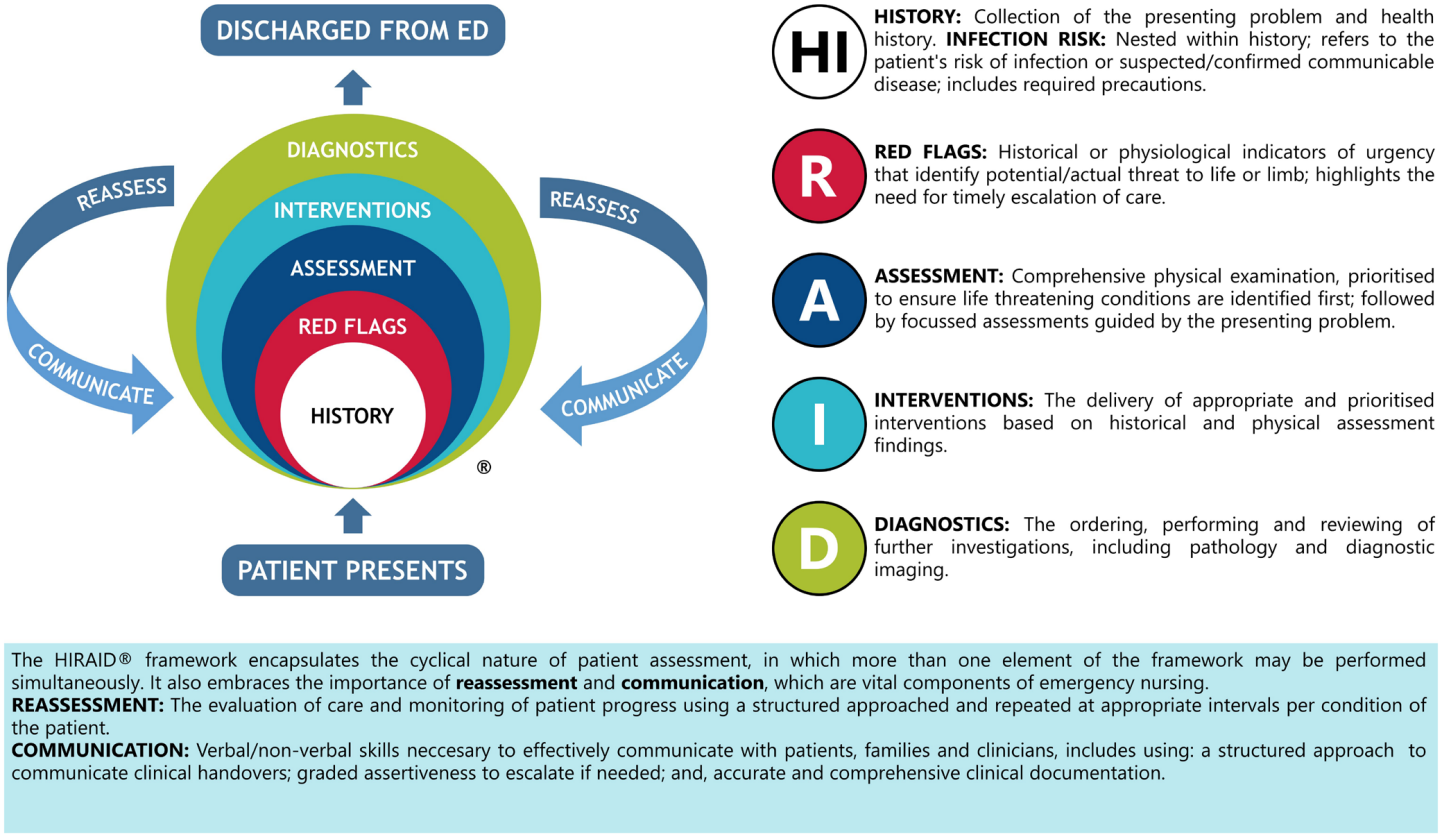
HIRAID emergency nursing assessment framework.

HIRAID is History including Infection risk, Red flags, Assessment, Interventions, Diagnostics, communication and reassessment (Munroe et al. 2015; Curtis et al. 2009) (Figure 1). HIRAID is a clinical safety system for use by emergency nurses for any patient presentation and the only validated framework designed to enable emergency nurses to systematically assess and manage emergency patients (Munroe et al. 2013). In feasibility, efficacy and replicability studies, implementation of the emergency nursing framework HIRAID reduced clinical deterioration related to emergency nursing care (Curtis, Munroe, et al. 2021), improved clinical documentation (Munroe et al. 2022) resulting in significant treatment cost savings (Curtis, Sivabalan, et al. 2021). Further, emergency nursing and medical staff reported that HIRAID improved the quality of clinical handover (Curtis et al. 2020).

To upscale HIRAID for use by more than 1300 registered nurses across 30 emergency departments in two Australian States (Curtis et al. 2023) we needed an effective, reliable, adaptable and replicable implementation design process and strategy. Successful implementation of any intervention in the emergency department is notoriously difficult due to workload unpredictability and high staff and patient turnover (de Wit et al. 2018; Jabbour et al. 2018). Although there has been a steady rise in the description of implementation methods, including educational strategies to introduce change in emergency departments, studies published to date have largely used observational methods and few are underpinned by a theoretical or evidence‐based framework (Jabbour et al. 2018; Tavender et al. 2016; Considine et al. 2023). Further, large interventional trials with a focus on implementation have failed to effectively change emergency nursing practice (Middleton et al. 2019; McInnes et al. 2020). Successful and sustained implementation of any intervention in complex healthcare settings requires consideration of clinician capability, motivation and opportunity (Curtis et al. 2017; Michie, Atkins, and West 2014).

This study adds to the literature by using theoretical frameworks to identify barriers and enablers to the implementation of HIRAID within a planned step‐wedge cluster randomised control trial (Curtis et al. 2023). During control conditions behavioural diagnostics were collected, to generate the output of this study—a data‐driven, behavioural‐focused implementation strategy. The implementation strategy needed to be adaptable to geographically and resource‐diverse emergency departments.

## 2 Aim

To develop a theory‐informed behaviour change‐driven strategy to maximise implementation and uptake of HIRAID in 30 Australian rural, regional and metropolitan emergency departments.

## 3 Design

**FIGURE 2:**
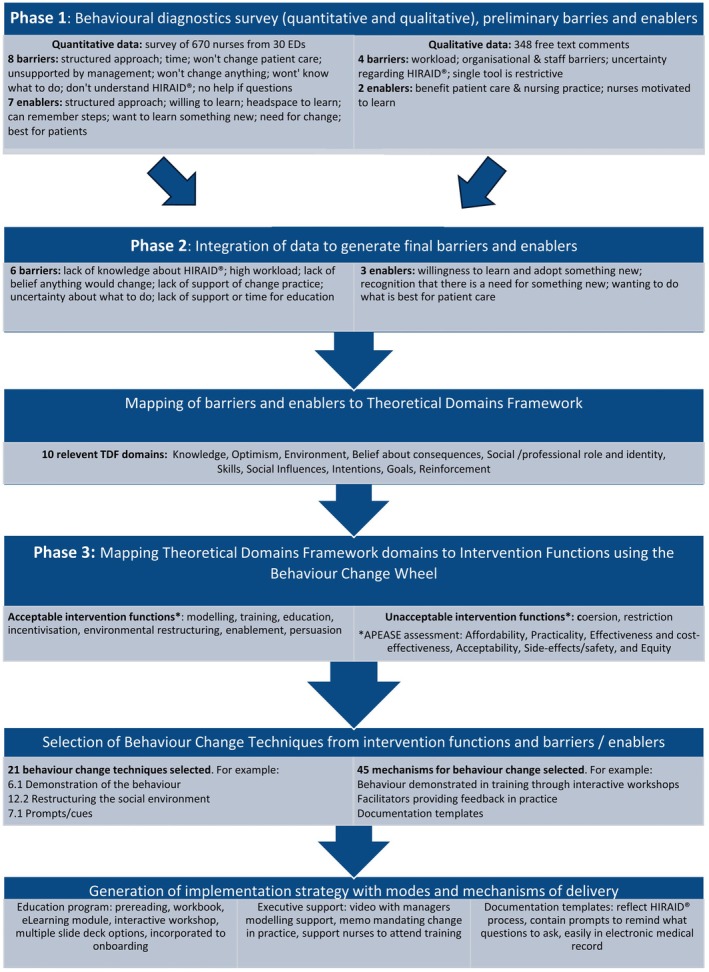
Summary of HIRAID implementation strategy development process and results.

There were three phases to this embedded mixed methods study (Creswell and Piano 2011) informed by the behaviour change wheel (Michie, Atkins, and West 2014) (Figure 2). First, concurrent quantitative and qualitative data were collected via an electronic survey, analysed to identify preliminary barriers and enablers to HIRAID implementation. Second, integration of Phase 1 findings identified overarching enablers and barriers to HIRAID implementation which were mapped to the domains of the Theoretical Domains Framework. Third, the selection of intervention functions and modes of delivery using the Behaviour Change Technique Taxonomy enabled the development of the HIRAID implementation strategy.

## 4 Methods

### 4.1 Clusters, Randomisation and Participants

Thirty NSW and Victorian emergency departments were grouped into four clusters based on geography and existing clinical governance systems (Table 1). Each cluster was randomised to commence the trial in the control condition and sequentially cross over to the intervention condition (implementation of HIRAID). The sites represent a range of geographically and clinically diverse settings, from large teaching hospitals to rural services where nurses call medical officers in when needed. Permanently employed emergency nurses—full‐time or part‐time, identified by nursing management at each site, were eligible to participate.

**TABLE 1:** Trial clusters, ED presentations, admissions and nursing staff.

Cluster	Clusters	ED patients per year	Admits via ED per year	ED Nurse staffing	Description of LHD
Cluster1	11 rural, regional EDs	116,836	17,065	188	Spans 44,534 km^2^ 200,000+ residents
Cluster2	12 rural, regional EDs	213,307	40,539	385	Spans 20,732 km^2^ 300,000+ residents
Cluster3	4 metro EDs	202,516	67,975	394	Spans 780 km^2^ 1.1+ million residents
Cluster4	3 metro EDs	169,465	47,320	410	Spans 2816 km^2^ 750,000+ residents
Total	30 EDs	702,124	172,899	1377	Range of remote, rural, regional and metro EDs

Abbreviations: ED, emergency departments; km^2^, square kilometre; LHD, local health district.

### 4.2 Phase 1: Behavioural Diagnostics Electronic Survey (Quantitative and Qualitative Data)

Presite visits and steering committee meetings informed the cross‐sectional survey design. The first section of the survey collected participant characteristics (place of work and years of nursing experience). We did not collect participant age and sex for all clusters as we did not plan to target male, female or nonbinary staff any differently in our intervention. We reported levels of experience rather than age, as this is more clinically relevant (Pinney et al. 2023). The 21‐item behavioural diagnostics section of the survey was used to identify barriers and enablers to HIRAID implementation. The questions for this electronic survey can be found in Supporting Information S2: Table 3. We used the Theoretical Domains Framework, which is a synthesis of behavioural change theories that applies the science of intervention implementation in health care to effect change (Atkins et al. 2017). The Theoretical Domains Framework was selected to enable targeted questions related to practice within the clinical environment, classification of barriers and enablers using a broad range of behavioural influences, and, as the investigators had previously successfully used the Theoretical Domains Framework to identify and design interventions in the emergency department/acute care context (Curtis et al. 2020; Kourouche et al. 2019; Murphy, McCloughen, and Curtis 2019). All 14 known domains of influence on behaviour were assessed, including *Knowledge*, *Skills*, *Reinforcement*, *Memory*, *Social Influences*, *Environmental Context/Resources*, *Beliefs about Consequences*, *Optimism*, *Goals*, *Social/Professional role and identity*, *Beliefs about capabilities*, *Intentions*, *Emotion and Behavioural regulation* (see Table 3 for domain definitions). Each section had free text options to further explain responses (Supporting Information S1).

Study data were collected and managed using REDCap (Research Electronic Data Capture) a secure, web‐based software platform and electronic data capture tool hosted at the University of Sydney (Harris et al. 2009). The survey was tested for face validity during a pilot phase and minor changes to formatting and flow were made. Surveys, with participant information and consent forms clearly stating the voluntary nature of the survey and anonymity of survey responses, were distributed to all eligible staff at each cluster by departmental managers and kept open for 4 weeks. Reminders were sent weekly. An incentive was offered to participants for completing surveys (a variety of 20 Australian dollar vouchers suitable for a participant's location, e.g., a local cafe) as a key strategy known to increase response rates (Abdelazeem et al. 2023). Data were managed per the 2018 National Health and Medical Research Council Australian code for the responsible conduct of research.

### 4.3 Phase 2: Integration of Findings to Generate Final Barriers and Enablers and Mapping of Barriers and Enablers to Theoretical Domains Framework

Prior to the integration of quantitative and qualitative findings, preliminary barriers and enablers were identified from the quantitative and qualitative survey data. This was to enable identification of any outlying sites, or variance by cluster. Barriers were defined as negatively worded statements with greater or equal to 80% agreement. Quantitative items were considered enablers if positively worded with greater or equal to 80% agreement with a statement (Amemori et al. 2011; Murphy, Curtis, and McCloughen 2019). Themes derived from content analysis (Graneheim and Lundman 2004) were considered barriers or enablers based on negatively or positively worded statements. The preliminary barriers and enablers were then integrated to form the final barriers or enablers. The final barriers and enablers were then allocated to the most appropriate domain of the Theoretical Domains Framework (Cane, O'Connor, and Michie 2012). Any items that crossed multiple domains were resolved through discussion among the research team, and the most relevant domain selected.

### 4.4 Phase 3: Selection of Intervention Functions and Behaviour Change Techniques to Generate HIRAID Implementation Strategy

Intervention functions for the relevant Theoretical Domains Framework domain were selected using the behaviour change wheel (Michie, Atkins, and West 2014) by mapping each of the Theoretical Domains Framework domains to the intervention functions. Corresponding behaviour change techniques to target the identified barriers and enablers were selected from the Behaviour Change Techniques Taxonomy (BCTT) (Michie, van Stralen, and West 2011). The following possible modes of delivery were also considered: content (what is to be delivered); provider (who is to deliver it); format (how was it delivered); setting (where is it to be delivered); recipient (to whom is it to be delivered); intensity (over how many contacts is it to be delivered) and duration (over what period of time) (Davidson et al. 2003). As not all recommended interventions, policies, behaviour change techniques and modes of delivery may be feasible or appropriate to deliver in different contexts, each intervention function and behaviour change technique was assessed using the APEASE criteria (Affordability, Practicability, Effectiveness and cost‐effectiveness, Acceptability, Side‐effects/safety and Equity) (Michie, Atkins, and West 2014) before inclusion in the implementation strategy.

### 4.5 Data Analysis

Quantitative data were extracted from REDCap and imported into SPSS Version 28 (IBM Corp 2013) for analyses. Numeric data were analysed descriptively using median and interquartile range (IQR) due to the skewed distribution, while categorical data were analysed using counts and percentages. The association between implementation sites and variables of interest, namely staff characteristics, behavioural analysis and use of HIRAID were examined using Chi‐square test (for categorical variables) and the Kruskal–Wallis *H* test (for numeric variables). The overall test was conducted with a significance level of 0.05. Pairwise comparisons were performed as needed.

Qualitative data obtained from the free text sections of the surveys were imported into NVivo v1 and analysed collectively using the Graneheim (Graneheim and Lundman 2004) method of inductive content analysis. The text was read several times to obtain a sense of the whole; the content was divided into meaningful units (codes) for analysis. The codes were abstracted into subthemes and themes at the highest level.

To facilitate integration, findings from each phase were merged in a table to simultaneously array the quantitative and qualitative results and assess for the ‘fit’ of the data (Fetters, Curry, and Creswell 2013). That is the coherence of the quantitative and qualitative findings. The data were assessed for as follows: *Confirmation*—that is, do findings from both types of data confirm the results of the other; *Expansion—*when findings from the two sources of data diverge and expand insights of the phenomenon of interest by addressing different or complimentary aspects of the phenomenon of interest and; *Discordance*—the qualitative and quantitative findings are inconsistent, incongruous or contradict each other (Fetters, Curry, and Creswell 2013). The quantitative and qualitative findings were integrated to form final barriers and enablers to HIRAID implementation.

### 4.6 Ethics Approval and Consent to Participate

Ethics approval was obtained for New South Wales sites through Greater Western Human Research Ethics Committee (2020/ETH02164) (approved October 2020), and for Victoria through Royal Brisbane & Woman's Hospital Human Research Ethics Committee (2021/QRBW/80026) (approved September 2021). Staff member indicated their consent using the online form. Responses were anonymous and the research team unable re‐identify participants from their responses.

## 5 Results

### 5.1 Phase 1: Behavioural Diagnostics Electronic Survey (Quantitative and Qualitative Data)

All clusters (*n* = 4) and all sites (*n* = 30) were represented by nursing staff who completed the survey (58% response rate). Respondents had an overall median of 5 (IQR [2–12] years of emergency nursing experience; Table 2). There was a statistically significant difference between clusters and years of emergency nursing experience (*H*(3) = 66.9, *p* < 0.001). Pairwise comparisons demonstrated nurses from rural/regional sites had statistically significant higher levels of experience compared to metropolitan sites (*d*(4 [2–8] and 5 [1–11] years for clusters 3 and 4 respectively *p* < 0.001)).

**TABLE 2:** Respondent characteristics, preliminary quantitative barriers and enablers to HIRAID implementation.

	Cluster1 (*n* = 102)	Cluster2 (*n* = 198)	Cluster3 (*n* = 148)	Cluster4 (*n* = 222)	ALL (*n* = 670)	Statistics*
Respondent characteristics *n* (%)						
Years of nursing experience, median [IQR]	16 [7–30]	14 [6–25]	6 [3–12]	8 [3–15]	**9 [4–19]**	**KW**_**3**_ **= 75.44, *p* < 0.001**
Years of emergency nursing, median [IQR]	8 [3–16]	8 [2–15]	4 [2–8]	5 [1–11]	**5 [2–12]**	**KW**_**3**_ **= 24.51, *p* < 0.001**
Highest qualification post nursing, *n* (%)						***χ***_**29**_ **= 29.0, *p* < 0.001**
None	31 (30.4%)	57 (28.8%)	76 (51.4%)	103 (46.4%)	**267 (39.9%)**	
Graduate certificate	41 (40.2%)	87 (43.9%)	40 (27.0%)	74 (33.3%)	**242 (36.1%)**	
Graduate diploma	18 (17.6%)	27 (13.6%)	14 (9.5%)	24 (10.8%)	**83 (12.4%)**	
Master's degree	12 (11.8%)	27 (13.6%)	18 (12.2%)	21 (9.5%)	**78 (11.6%)**	
What are the best ways for you to learn about how to do something new? *n* (%)						
Face‐to‐face education	97 (95.1%)	182 (91.9%)	140 (94.6%)	208 (93.7%)	**627 (93.4%)**	*χ*_23_ = 1.56, *p* = 0.669
Opportunity to ask questions	62 (60.8%)	139 (70.2%)	107 (72.3%)	156 (70.3%)	**464 (69.2%)**	*χ*_23_ = 4.27, *p* = 0.234
Feedback from my manager or educator about how I was performing	50 (49.0%)	124 (62.6%)	104 (70.3%)	114 (51.4%)	**392 (58.6%)**	***χ***_**23**_ **= 18.28, *p* < 0.0001**
Online learning	49 (48.0%)	68 (34.3%)	52 (35.1%)	80 (36.0%)	**249 (37.1%)**	*χ*_23_ = 6.22, *p* = 0.101
Hands‐on practice	87 (85.3%)	187 (94.4%)	134 (90.5%)	202 (91%)	**610 (91.1%)**	*χ*_23_ = 6.99, *p* = 0.072
Behavioural analysis—percentage agreement with the following statements *n* (%)						
Preliminary barriers						
I don't understand what HIRAID is	24 (23.5%)	87 (43.9%)	11 (7.4%)	141 (63.5%)	**263 (39.3%)**	***χ***_**23**_ **= 130.04, *p* < 0.0001**
There is not enough time to change the way of working	25 (24.5%)	61 (30.8%)	67 (45.3%)	88 (39.6%)	**241 (35.9%)**	***χ***_**23**_ **= 14.96, *p* = 0.002**
Nothing will change	28 (27.5%)	50 (25.3%)	51 (34.5%)	77 (34.7%)	**206 (30.7%)**	*χ*_23_ = 5.90, *p* = 0.126
It won't change the way I care for my patient	22 (21.6%)	40 (20.2%)	56 (37.8%)	54 (24.3%)	**172 (25.6%)**	***χ***_**23**_ **= 15.70, *p* = 0.001**
I am worried I won't know what to do	17 (16.7%)	35 (17.7%)	39 (26.4%)	65 (29.3%)	**156 (23.2%)**	***χ***_**23**_ **= 11.23, *p* = 0.011**
Change is unsupported by management	18 (17.6%)	49 (24.7%)	37 (25%)	42 (18.9%)	**146 (21.8%)**	*χ*_23_ = 4.01, *p* = 0.260
I am worried no one will help me with questions when I try and use it	22 (21.6%)	38 (19.2%)	32 (21.6%)	47 (21.2%)	**139 (20.7%)**	*χ*_23_ = 0.43, *p* = 0.935
Preliminary enablers						
I want to do what is best for patient care	94 (92.2%)	184 (92.9%)	143 (96.6%)	212 (95.5%)	**633 (94.3%)**	*χ*_23_ = 3.71, *p* = 0.290
Are you willing to learn and adopt something new?	93 (91.2%)	183 (92.4%)	130 (87.8%)	195 (87.8%)	**601 (89.7%)**	*χ*_26_ = 6.10, *p* = 0.412
A structured approach is beneficial (B/E)	87 (85.3%)	156 (78.8%)	116 (77.9%)	165 (74.3%)	**524 (78.1%)**	*χ*_26_ = 6.28, *p* = 0.392
The way we do things is fine no need to change anything	7 (6.9%)	24 (12.1%)	24 (16.2%)	34 (15.3%)	**89 (13.3%)**	*χ*_23_ = 5.78, *p* = 0.123
I don't have the headspace to learn something new	4 (3.9%)	9 (4.5%)	15 (10.1%)	37 (16.7%)	**65 (9.7%)**	***χ***_**23**_ **= 22.22, *p* < 0.0001**
It's too hard to remember anything new	1 (1%)	5 (2.5%)	13 (8.8%)	18 (8.1%)	**37 (5.5%)**	***χ***_**23**_ **= 13.30, *p* = 0.004**
I don't want to learn something new	1 (1%)	5 (2.5%)	8 (5.4%)	5 (2.3%)	**19 (2.8%)**	*χ*_23_ = 5.17, *p* = 0.160

Abbreviations: (B), barrier; (E), enabler; ED, emergency department; IQR, interquartile range.

* Results in bold are statistically significant.

**FIGURE 3:**
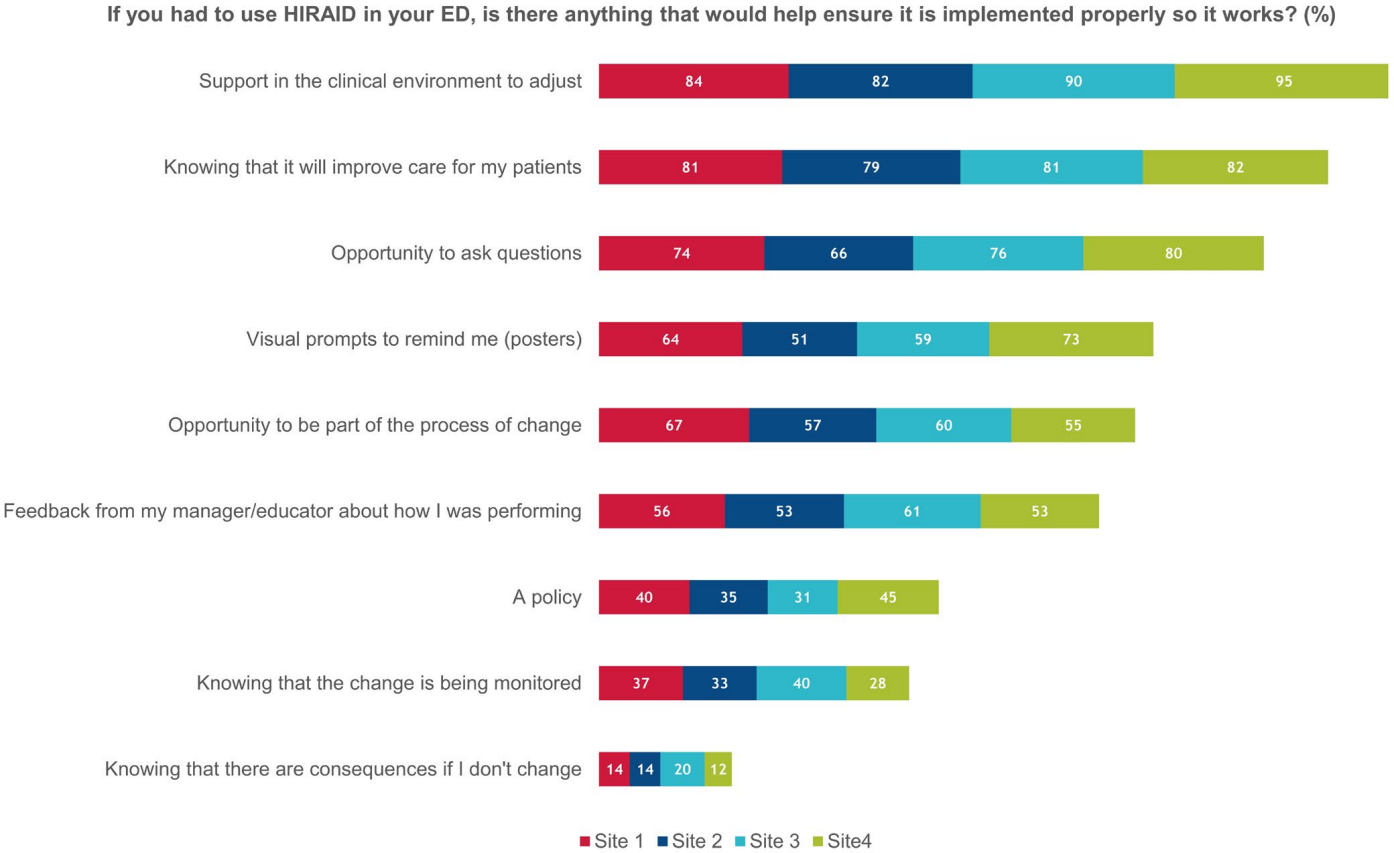
Quantitative responses for what would help implementation of HIRAID.

**FIGURE 4:**
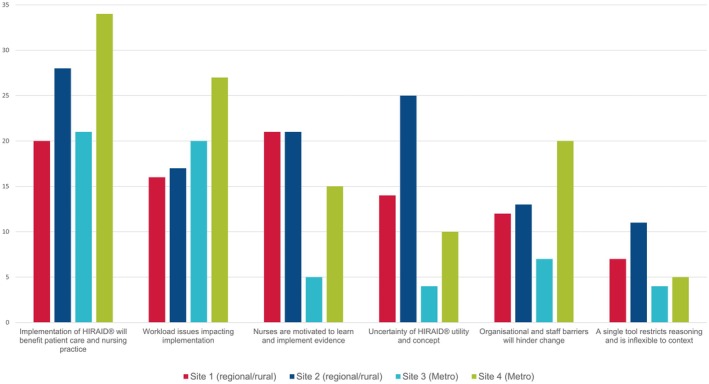
Themes generated from qualitative data per cluster.

Respondents indicated the best ways for them to learn something new was through face‐to‐face education (93.4%), hands‐on practice (91.1%) and the opportunity to ask questions (69.2%) (Table 2). The only outlier to this finding were respondents from Cluster 3, who indicated that feedback from their manager or educator on how they were performing was helpful (70.3%); nurses at this cluster were also the least experienced (Figures 3 and 4).

There were seven enablers and seven barriers identified from the quantitative data (Table 2). Enablers at all clusters were that respondents agree they want to do what is best for patient care (94.3%), willing to adopt something new (89.7%). Although the overall percentage agreement that a structured approach to patient care is beneficial was only 78.1%, one cluster recorded 85.3%, resulting in this being considered a barrier and an enabler (Cluster1, Table 2). Most did not think the way they do things in their emergency department was fine (86.7%) but were concerned they would not know what to do with HIRAID (23.2%), what HIRAID was (39.3%) or that nothing would change (30.7%). More than a third (35.9%) of respondents felt there was not enough time to change their ways of working, particularly in the largest metropolitan cluster (45.3%; Cluster3, Table 2).

The qualitative survey data yielded 348 free text comments that generated 359 codes. The codes led to the construction of six themes and 21 subthemes. All clusters were represented in each theme (Figure 4; Supporting Information S2: Table 1). The themes were categorised as enablers or barriers. Two enablers and four barriers were identified.

The most prominent theme was an enabler. Respondents overwhelmingly felt the implementation of HIRAID would benefit patient care and nursing practice (*n* = 106). Nurses reported they were motivated to support the intervention as it was evidence‐based (*n* = 62) and were willing to learn new things (*n* = 22), *‘excited about change and improvement of practice. I am excited to learn new things’ (Cluster3, P4)*. Some were more pragmatic *‘The current system is very broken. Anything that might help to improve it is worth trying’ (Cluster4, P86)*. Respondents highlighted the impact HIRAID may have for junior or casual staff, ‘*A structure that is consistent for all would be beneficial and beneficial for new staff’ (Cluster2, P63)* and *‘It would provide confidence to all staff, but especially newer emergency department nurses’ (Cluster1, P87)*. In contrast, there were some respondents who felt a single tool is restrictive (*n* = 27) and may not suit their emergency department (*n* = 15).

The second most common theme was a barrier and reflected concern that HIRAID implementation might increase their workload (*n* = 88), for example, *‘I think most people would be willing to learn something new. However if the new process is more time consuming/means less face to face time with patients and more time standing at the computer doing notes there will be a lack of compliance’ (Cluster4, P193)*. Another concern emphasised organisatonal barriers to implementation (*n* = 69), in particular, a lack of protected time or support for education (*n* = 24) *‘No support from CNUMS [Clinical Managers], educators or NUM [Nurse Unit Managers] So can't learn with no support and being smashed with patient load and negativity all around you’ (Cluster3, P67)*. Organisational culture was also raised as a barrier to change *‘The implementation needs ALL to cooperate, not just a committed few’. (Cluster1, P65)*. Some respondents were uncertain of the utility and benefits of HIRAID (*n* = 58), *‘Any new approach may or may not be beneficial. Need to know more to see if this is adaptable to a small, poorly staffed cluster. Willing to try’ (Cluster2, P17)*.

### 5.2 Phase 2: Integration of Phase 1 Findings to Generate Final Barriers and Enablers and Mapping of Barriers and Enablers to Theoretical Domains Framework

In Phase 1, 12 preliminary barriers (seven quantitative and four qualitative) and nine preliminary enablers (seven quantitative and two qualitative) to HIRAID implementation were identified. When quantitative and qualitative findings results were integrated, there were six final barriers and three enablers (Supporting Information S2: Table 2).

The final six barriers were (i) lack of knowledge about HIRAID; (ii) an already high workload that would be increased, (iii) lack of belief anything would change; (iv) HIRAID not suitable for the workplace; (v), uncertainty about what to do; and (vi) lack of support to attend education or implement change. The three enablers to HIRAID implementation were as follows: (i) willingness to learn and adopt something new; (ii) recognition of the need for something new and (iii) wanting to do what is best for patient care. There was minimal discordance between the quantitative and qualitative results. The merging of quantitative and qualitative findings was confirmatory and resulted in expanded barriers and enablers with more meaning. For example, the quantitative results indicated that lack of knowledge was an issue at only three of the clusters; however, the qualitative results highlighted misunderstanding of what HIRAID is at the fourth cluster, and thus lack of knowledge was added as a barrier for the fourth cluster.

One of the strongest barriers was a high workload and perception that implementing change (HIRAID) would increase this workload. Just over a third of respondents (35.9%) felt there was not enough time to change the way of working. This proportion was larger (45.3%) in Cluster 3 (a large metropolitan site with the most presentations per year) *‘…the main hurdle introducing HIRAID to a Regional ED [emergency department] is TIME to document, which requires adequate staffing Levels and I can't see that ever changing as we are currently at breaking point already with the workload/expectations placed on us’ (Cluster1, P16)*. Although this survey was conducted during the COVID‐19 pandemic, there was very little mention of this in responses, but change fatigue was apparent, *‘There has been substantial change over the past years—even during COVID—we are all mentally exhausted. The pressures have been relentless’ (Cluster4, P99)* and *‘If it requires significant time to develop new skills to assess patients this will only contribute more to staff stress level—we literally do not have the time on the floor to start over again so if it isn't quick and easy then it will be hard’ (Cluster4, P116)*.

Almost a third of respondents (30.7%) felt nothing would change, and a quarter (25.6%) that HIRAID would not change the way they cared for their patient. Again, this was most reported in Cluster 3 (37.8%). *‘I would like to know the reasons WHY the change is being made and how it will improve things. If this information is not provided I will be less inclined to try and learn a new system’ (Cluster3, P132)*.

A lack of support to implement change in clinical practice and attend/receive education was of great concern for respondents. Nearly a quarter (represented at all 30 sites) felt unsupported by management, more so in the Clusters 2 and 3. *‘We have grossly inadequate staffing, no permanent management team on site, no educator at the facility for over 12 months and increasing lack of medical officers with expected scope of practice with no resources or remuneration’ (Cluster1, P10)*. Further, they were worried no one will help answer questions in the clinical environment (20.7%). These concerns were strongly reflected in qualitative responses *‘I wont be given enough time or practice to learn anything new before it is implemented and subsequent changes (eg like in EMR) get sent through on a generic email, no education etc. … no budget is a feature of NSW Health “implementation” strategies …’ (Cluster2, P42)*.

The three enablers to HIRAID implementation were as follows: (i) willingness to learn and adopt something new; (ii) recognition of the need for something new; and (iii) wanting to do what is best for patient care. More than 89.7% of respondents were willing to learning something new, exemplified by the high number of respondents motivated to learn and implement evidence (*n* = 62) *‘Excited about change and improvement of practice. I am excited to learn new things’ (Cluster3, P4)*. This was consistent across all clusters. Further, only 13.3% of respondents felt there was no need to change anything, and, most felt a more structured approach would be beneficial (78.1%). *‘There is far too much variability in care provided at present, it can be a game of lotto when it comes to the patient experience with nurses’. (Cluster4, P86)*. Respondents from some of the smaller sites were less convinced that change was required, however, identification of this reluctance was very valuable as it enabled targeted selection of strategies.

The final barriers and enablers were mapped to 10 domains of the Theoretical Domains Framework: Knowledge, Optimism, Environment, Belief about consequences, Social/professional role and identity, Skills, Social Influences, Intentions, Goals, Reinforcement (Table 3; Supporting Information S2: Table 2).

**TABLE 3:** Barriers and enablers to HIRAID implementation mapped to behaviour change wheel intervention functions, and behaviour change techniques (BCTs) to form HIRAID implementation strategy.

Theoretical domains (*and definition*)	Barriers (B) and Enablers (E) to be addressed	Intervention functions (*and definition*)	Behaviour change techniques, Modes of delivery (in bold) and intervention content
Capability—an individual's psychological and physical ability to participate in an activity			
**Knowledge** *An awareness of the existence of something*	Poor understanding of what HIRAID is, how it is used and why (B)	**Education** *Increasing knowledge and understanding by informing, explaining, showing and correcting*	*5.1 Information on health consequences* ‐ Provide staff with information on health consequences of using HIRAID within the **education programme** (instructor/participant), for example, research results from the evaluation of HIRAID, including, decreased adverse events ‐ Develop and distribute a **participant workbook** with information on HIRAID *5.3 Information about social and environmental consequences* ‐ Provide staff with information about HIRAID impacts on emergency nursing care, e.g., improved documentation, and self‐confidence in the **education programme**
**Skills** *An ability or proficiency acquired through practice*	Unsure of what to do (B)	**Training** *Increasing psychological or physical skills, or habit strength by explanation, demonstration, practice, feedback and correction*	*4.1 Instruction on how to perform behaviour* ‐ **Video** instructing on how to use HIRAID in practice in **eLearning module** *6.1 Demonstration of the behaviour* ‐ Behaviour demonstrated in training through **interactive workshops** *8.1 Practice rehearsal* ‐ In **interactive workshops**, demonstrate A to G assessment, then ask participants to perform reassessment, and then give the group feedback *8.3 Habit formation* ‐ Adoption of the HIRAID framework into broader ED education to increase nurse exposure to relevant application of skill and allows for ongoing training
Opportunity—refers to external factors that make a behaviour possible			
**Environmental context and resources** *Any circumstance of a person's situation or environment that discourages or encourages the development of skills and abilities, independence, social competence and adaptive behaviour*	Lack of support or time for education (B) Implementation aided by Face‐to‐face education and hands‐on practice (E)	**Environmental restructuring** *Constraining or promoting behaviour by shaping the physical or social environment*	*7.1 Prompts/cues* ‐ Integrate documentation templates into the electronic medical record system to support and reduce cognitive load *12.2 Restructuring the social environment* ‐ Provide clinical supervision ‐ **Champions/educators** providing feedback in practice, addressing staff where areas for improvements and acknowledging where done well ‐ Provide protected time to complete the education programme including the eLearning ‐ **Face‐to‐face interactive workshops** as part of the education programme ‐ In **shift huddles** using inclusive language provide feedback to staff on behaviour in using HIRAID from shift/day before ‐ Regular **stakeholder meetings** to discuss implementation readiness and plans, and to troubleshoot problems ‐ Recruit and train local clinical leaders in the use of HIRAID in instructor courses (Train the trainer) *11.3 Conserving mental resources* ‐ Provide **flip cards** to support recall framework steps
**Social influences** *Interpersonal processes that can cause individuals to change their thoughts, feelings, or behaviours*	Lack of support (B)	**Enablement** *Providing support to improve ability to change in a variety of ways not covered by other intervention functions*	*6.3 Information about others approval* ‐ Ensure management support is evident through support education time, meetings/huddles, access to data and change in practice. In the provider course, discuss the need to assess the patients using the HIRAID framework ‐ Provide go‐to statements/catch phrases to be used by champions/staff to assist in reframing the behaviour (activities plan) ‐ Email from hospital executives in support of HIRAID, and showing support ‐ Peer support through a video and electing to be champions ‐ Management support in video and show of support by giving time for study days/HIRAID training *3.2 Social support (practical)* ‐ Support from educators to allow staff to take time to complete necessary assessment and documentation to increase familiarity and competency ‐ Facilitation by HIRAID CNCs including site visits, and tracking of site education and issues ‐ **Daily walk‐through ED** by core research nurse/HIRAID champion/Educator to monitor, answer questions, assist, praise, feedback ‐ Ongoing support from research team in education, implementation and stakeholder meetings
Motivation—refers to the conscious and unconscious cognitive processes that direct and inspire behaviour			
**Optimism** *The confidence that things will happen for the best or that desired goals will be attained*	Lack of time and high workload (B)	**Persuasion** *Changing the way people feel about behaviour by generating cognitive dissonance and showing how changing behaviour can reduce it* **Modelling** *Showing examples of the behaviour for people to imitate*	*6.3 Information about others approval* ‐ Demonstrate leadership support for implementation, e.g., memo from hospital executives to nursing staff supporting the project, and commitment of work time to complete education ‐ Feature senior staff and relevant team members in a **video** showing their approval *6.2 Social Comparison* ‐ Demonstrate to staff how implementation has been achieved at other sites
**Belief about consequences** *Acceptance of the truth, reality, or validity about outcomes of a behaviour in a given situation*	Lack of belief that change will happen (B)	**Education** **Persuasion**	*15.1 Information on health consequences* ‐ Provide staff with information on health consequences of using HIRAID within the **education programme** (instructor/participant), for example, research results from the evaluation of HIRAID, including, decreased adverse events ‐ Develop and distribute a **participant workbook** with information on HIRAID *5.3 Information about social and environmental consequences* ‐ Provide staff with information about HIRAID impacts on emergency nursing care, e.g., improved documentation, and self‐confidence in the **education programme** *1.4 Action planning* ‐ Implementation plan for each site, including who is making the changes, and timeframes and milestones, and progress measures
**Social/professional role and identity** *A coherent set of behaviours and displayed personal qualities of an individual in a social or work setting*	Belief intervention will not change the way they work (B) Need to change way of working (E)	**Persuasion** **Modelling**	*13.2 Framing/reframing* ‐ Ensure experienced staff understand that it does not necessarily change work practice but provides common terminology and approach to assessment in the emergency department (**Education programme)** *15.1 Verbal persuasion about capability* ‐ Trainers develop phrases what they support/encourage staff use of HIRAID delivered/developed in the train the trainer education *6.1 Demonstration of the behaviour* ‐ Instructors/trainers/champions demonstrate behaviour in clinical practice and emergency department education ‐ HIRAID CNCs encouraged to attend train the trainer sessions at other clusters
**Intentions** *A conscious decision to perform a behaviour or a resolve to act in a certain way*.	Nurses willing to learn and adopt something new (E)	**Modelling**	*13.1 identification of self as a role model* ‐ Staff self‐nominate as champions, thereby identifying themselves as role model
**Goals** *Mental representations of outcomes or end states that an individual wants to achieve*.	Evidence for intervention will support implementation as want to do what is best for patient care (E)	**Persuasion**	*9.1 Credible source* ‐ HIRAID video included ED nurse leaders from across the district as well as ED staff from clusters supporting HIRAID ‐ Provide information on evidence and development of education programme and implementation plan. For example, the educational programme was designed using educational theory to scaffold learning and build on existing knowledge and included varied delivery methods to cater to different learning styles. The implementation plan was developed using behaviour change theory ‐ In interactive **workshops**, provide examples of poor documentation and how it has improved since using HIRAID at other sites *6.3 Information about others approval* ‐ Reporting of previous research and feedback from ED staff who use in practice, included in the instructor training. Instructors are encouraged to use this information with messaging to support/encourage application in practice
**Reinforcement** Increasing the probability of a response by arranging a dependent relationship, or contingency, between the response and a given stimulus	Support in the clinical environment helps implementation (E)	**Environmental restructuring** **Incentivisation** Changing the attractiveness of a behaviour by creating the expectation of a desired outcome or avoidance of an undesired one	*2.2 Feedback on behaviour* ‐ CNCs/Educators/champions/research nurses to provide regular feedback to staff individually in coaching during patient care. Praise for a job well done and highlight areas to improve ‐ Champions/educators provide feedback in practice, support application and documentation ‐ Managers, leadership praise when HIRAID is used, positive reinforcement ‐ Audit—audit conducted 6/12 weeks during implementation—feedback aggregate on use per site *2.7 Feedback on outcomes of behaviour* ‐ Interactive feedback provided based on responses in interactive case studies in eLearning and interactive workshops *7.5 Remove aversive stimulus* ‐ Remove old documentation templates *10.1 material incentive* ‐ Monitor uptake through HIRAID templates, those consistently using and sites with the greatest proportion will have opportunity to receive prizes' varied by site based on local input

Abbreviations: (B), barrier; BCT, behaviour change technique; CNC, Clinical Nurse Consultant; (E), enabler; HIRAID, History, including Infection risk, Red flags, Assessment, Interventions, Diagnostics, communication and reassessment, Definitions of intervention functions (Cane, O'Connor, and Michie 2012).

### 5.3 Phase 3: Selection of Intervention Functions and Behaviour Change Techniques to Generate HIRAID Implementation Strategy

The 10 Theoretical Domains Framework domains were mapped to nine intervention functions of the behaviour change wheel: modelling, training, education, incentivisation, environmental restructuring, enablement, persuasion, coercion and restriction (Supporting Information S2, Figure 1). Coercion and restriction were deemed not suitable after application of the APEASE criteria acceptability, equity and safety, leaving the seven final intervention functions.

The seven intervention functions were mapped to 21 behaviour change techniques after APEASE criteria review. These were then operationalised with 45 implementation strategies through modes of delivery such as a video, education sessions, visual prompt for electronic medical records and change facilitators. A summary of the proposed intervention functions, policy and behaviour change techniques to be used to overcome modifiable barriers and enhance enablers in the implementation of HIRAID is provided in Table 3. The most significant components of the strategy were the education programme, executive support and modifications to nursing documentation procedures.

To support the behaviour change interventions, ameliorate barriers and enhance the learning experience the HIRAID learning resources (prereading, participant workbook, e‐learning module and facilitated interactive workshop) were developed using the educational principles of constructive alignment (Biggs and Tang 2010, 2015) backwards design (Davila Rubio 2017) and scaffolded learning (Wood, Bruner, and Ross 1976). These outcomes‐driven approaches start with the intended learning outcomes: what participants should be able to do and what is the expected standard. Each component of HIRAID has detailed learning outcomes so instructors and participants have clarity about what they are trying to achieve. Educational experts purposefully designed the evidence‐informed education programme to put structure around many of the things emergency nurses already do. Drawing on best available evidence regarding patient assessment, risk factors for adverse outcomes, recognising and responding to clinical deterioration and educational pedagogy to develop deep learning of core concepts and high‐order thinking. The programme was also aligned with the College of Emergency Nursing Australasia Practice Standards for the Emergency Nursing Specialist (College of Emergency Nursing Australasia [CENA] 2020) and Australian Commission on Safety and Quality in Health Care (National Safety and Quality Health Service Standards) (Australian Commission on Safety and Quality in Health Care [ACSQHC] 2017).

Further, HIRAID Instructors were trained to implement HIRAID with a train‐the‐trainer model. As our nursing education workforce is increasingly less experienced, we also developed an instructor manual to provide a best practice framework to support participants in the development of knowledge, skills, behaviours and professional attributes necessary to apply the HIRAID framework in clinical practice (CENA 2020; ACSQHC 2017).

Demonstration of executive support was a key feature of the implementation strategy, for example, a video with managers, a memo from hospital executives to nursing staff supporting the project and a commitment of work time to complete education. Central to all implementation frameworks is executive support. High level, visible, formal support empowers nurse managers to enforce the change in practice, enables dedicated training time for staff and goes some way to mitigating the effects of micropolitics (Rogers et al. 2020).

Nursing documentation procedures were modified by integrating documentation templates into the electronic medical record system to support and reduce cognitive load. Accurate clinical documentation is a nursing professional responsibility essential for high quality and safe patient care (Ho et al. 2014). The healthcare record is one of the most important sources of patient information and is a legal record of care delivered that facilitates information exchange between health professionals (Ho et al. 2014). When compared to other implementation strategies in earlier HIRAID research, nurses reported documentation templates to be the most useful prompt in their clinical practice (Curtis et al. 2020) and resulted in significantly improved quality and quantity of information recorded about emergency patient care (Munroe et al. 2022).

A simplified and modifiable implementation plan was developed for frontline clinicians. This provides a summary of the strategies and proposed interventions; an action and explanation checklist; and considerations for context can be found in Table 4.

**TABLE 4:** Proposed implementation actions for effective implementation of HIRAID, including actions, explanations and considerations.

Proposed interventions	Actions/explanation	Considerations
Project implementation plan/checklist	Establish a project plan Include all the below items. Also, include a risk register and tracking system (a template can be provided)	‐ What is the context for implementation? ‐ Is the site ready for implementation? ‐ Who will lead the implementation?
HIRAID lead	Establish lead HIRAID nurse HIRAID lead should be an experienced RN with clinical credibility, teaching skills, education experience, organisational expertise, project management and change management skills	‐ Do you have funding? Is there an existing staff member with the capacity for dedicated time to lead? ‐ Are they familiar with context and emergency assessment?
Executive support	Meet with hospital executive regarding the process for implementation Memo from executive to all nursing staff about training and go live date (example can be provided) Demonstrate commitment from Hospital and Nursing Executive to implement HIRAID which includes staff support to attend training, will demonstrate support of implementation as a priority Meet with nurse managers to ensure support Nurse managers support to reinforce the practice change as well as interventions in this implementation strategy	‐ What is the best method for communication between the Hospital and the Nursing executive at the site? Would email be appropriate or face‐to‐face meetings with staff? ‐ How will the nurse manager communicate with their team? What works best at this site? e.g. shift huddles, team meetings, emails
Stakeholder engagement and management	Determine key individuals and groups HIRAID lead meets with senior nursing and medical staff from all sites in person and provides an overview of HIRAID and the background behind the framework (PPT can be provided) Identify appropriate meetings to attend and preferred process for communication HIRAID lead attends/reports to local ED, education, hospital quality and nursing exec meetings and has HIRAID implementation established as an agenda item to ensure ongoing support and monitoring for a 6‐month period	‐ Who are the key stakeholders for the site? ‐ What are the best methods for communication? ‐ Are there regular meetings set up? Consider establishing a steering committee to guide and support planning and implementation
Implementation support	Regular site visits or phone calls The HIRAID lead support sites with the HIRAID implementation, assisting the smaller sites visiting in person over consecutive days to assist with education and training, and available on call to provide guidance and support when required FAQ document (provided) A FAQ tailored to local context is circulated to answer common questions raised by staff. This provides consistent information to all sites	‐ Discuss with sites how they would prefer to be contacted
HIRAID instructor course	Booking of rooms and times Request expressions of interest for staff to be instructors Staff (instructors) rostered to attend from each site Identify local clinical incidents related to ED, as well as any examples of substandard documentation in ED to use in presentations The HIRAID Instructor course is a 4‐h^a^ course that teaches HIRAID and its application. The course includes case studies and activities to apply knowledge. Equips staff as role models, with the capacity to support and teach HIRAID. Relevant site‐specific information, identifying the need for change, benefits illustrated through previous research and feedback from nursing staff who have used HIRAID at other sites is incorporated into training. Instruction will be provided on feedback processes for the health district (i.e. auditing site compliance and education completion). Staff identified to attend the HIRAID Instructor course can self‐nominate or be nominated by Nurse unit manager/Educator	‐ Who would be instructors? Discuss with stakeholders ‐ Where will the programme be held?
HIRAID provider course + learning resources	Four phases of compulsory education, endorsed by the College of Emergency Nursing Australasia and Australian College of Nursing Pre‐reading: Chapter 13: Patient Assessment and Essentials of Care from *Emergency and Trauma Care for nurses and paramedics, 4th Edition* 2 The workbook: apply principals HIRAID to the scenario; it requires the individual to reflect reflection on local processes relevant to the application of HIRAID 3 eLearning (see below) 4 Face to face (see below) Establish a person responsible for sites and reporting mechanisms to the HIRAID lead Site educators and/or HIRAID lead will sight completed workbook and track staff completion of all education components—report to exec	‐ What will be the best way for site to run the sessions? e.g. could run as a half‐day session on 1 day or over a few days or weeks depending on staffing etc.
eLearning module	Set up eLearning module access 1.5‐h module with paediatric and adult scenarios aids in knowledge and skill development through demonstration of behaviour, with the ability to identify and prioritise the appropriate nursing actions. The module enables practice rehearsal through interactive design, adult learning principles and activities It can be done before or during the programme‐allocated time	‐ Can have dedicated staff time to complete, e.g. during in‐service or programme time? ‐ Will computers be available for staff to complete?
Inservice (short education) (part of provider)	A 1‐h session with interactive application of HIRAID principals delivered by emergency educators and clinical champions, who completed the HIRAID Instructor workshop. The education design allows for flexible delivery through either facilitated presentation of a case study or simulated delivery. The integration of role‐play in training will aid in increasing staff confidence and capacity using the HIRAID framework. The environmental changes (eMR templates, FLIP cards) with the rollout of HIRAID in clinical practice will be covered. The teaching session will reinforce the messages communicated by executives and managers related to the requirement that the framework be implemented in clinical practice. Staff will be informed of the audits and evaluations to be undertaken related to the HIRAID framework implementation Book times/rooms Recording of attendance to HIRAID lead	‐ What support will staff need to support attendance? ‐ How will Instructors keep records of attendance? ‐ How can the content be tailored? Eg if site does not have paediatric presentations, can do the two adult scenarios
Electronic medical record (eMR)	Local review of templates Compliance/monitoring and feedback process by nurse educators/managers HIRAID documentation templates will be incorporated into eMR, and all staff will be set up to have them available for documentation. The templates support mental recall with the provision of prompts for content Any previous documentation templates are removed from eMR	‐ How is the medical record used by staff? Is there a capacity for prompts to be built into the system? ‐ How will the process be streamlined?
Short comms (posters, emails)	Memo from nursing exec distributed by nurse managers (draft can be provided) Communication of staff expectation re training completion and use of HIRAID framework at executive and local level prior to going live date and during rollout. Expectations are reinforced locally via staff communication from managers through meetings, email communication and 1:1 communication. Communication will include audit information and follow‐up to occur to monitor use and processes for staff not using in practice Poster and/or Kiosk computer screen saver Posters/screen saver to communicate the commencement and remind staff of the expectation for it to be used in all ED clinical documentation	‐ What will be the best ways to communicate at the site? From who? ‐ What is the process for staff not compliant with change?
Tracking/monitoring	Audits at 6 and 12 weeks by lead Tracking system changes Track the completion of each component of education, use of documentation templates at 6 and 12‐week post‐implementation, system changes (eMR, screen savers, flip card, comms)	‐ What will be the best way to ensure the implementation process has been completed?
Local champions/modelling	Role expectations of instructors Availability for questions Modelling of HIRAID use in clinical practice through HIRAID Instructor course. HIRAID champions will support clinicians in skill development and encourage use in practice. HIRAID champions are provided with role instructions	—
Resources/prompts	Provide flip card for ID tag All emergency nurses are provided with flip cards outlining HIRAID to support recall and support use in documentation	—
Orientation process	HIRAID training is incorporated into ED orientation and includes eLearning, short in‐service and role‐play applications Instruction on the HIRAID templates, highlighting the difference in documentation to other clinical areas and the expected practice standard in the ED	‐ How will the implementation be sustained ongoing? How will new nurses be orientated? Whose responsibility will it be? ‐ HIRAID team liaise with LHD education teams

*Note*: Implementation strategies should be considered against the APEASE criteria (Affordability, Practicability, Effectiveness and cost‐effectiveness, Acceptability, Side‐effects/safety and Equity).

^a^ Assumed prior learning is completion of HIRAID Provider training.

## 6 Discussion

This multicentre study focused on identifying the barriers and enablers to emergency nurses implementing a new evidence‐based intervention to better support patient safety and outcomes. To optimise the implementation of the emergency nursing framework (HIRAID) at scale an implementation strategy was developed using a systematic process guided by the behaviour change wheel, which resulted in the generation of a multifaceted implementation strategy containing 22 behaviour change techniques adaptable for use across rural, regional and metropolitan emergency departments.

There was considerable variability in characteristics across the 30 emergency departments, with a range of different contexts, sizes and resources for the departments. The clusters varied from large metropolitan emergency departments to regional and smaller rural centres. Despite the range of contexts, most barriers and enablers were shared across clusters. An enabler—nurses were willing to learn something new if it is evidence‐based and improves patient care—was the most prominent finding of this study. This finding is consistent with international literature that reports nurses perceive learning is fundamental to professionalism, but motivation to learn is influenced by the perceived impact on practice and patient outcomes (King et al. 2021; Mlambo, Silén, and McGrath 2021; Brekelmans et al. 2016). We ensured that the evidence for HIRAID was clearly communicated and embedded in all modes of delivery in implementation strategy.

Despite the enthusiasm of emergency nurse respondents to implement evidence for the benefit of patients, time and workload barriers to implementation were dominant. The reduced capacity and motivation of nurses to change their practice due to workload not new (Cheraghi et al. 2023). Reluctance to change is influenced by a multitude of external (policies, resources and incentives) and internal (local organisational readiness, infrastructures and workflows) contextual characteristics (Cheraghi et al. 2023; Weiner and Hoppe 2023). Another barrier, especially at the nonmetropolitan clusters was a lack of support in the clinical environment to assist implementation. Considerations for implementation at the regional and rural clusters needed to particularly consider the variability in available clinical services and staffing, with many of the smaller emergency departments without 24‐h medical coverage (Smith et al. 2021). Further, although this survey was conducted during the COVID‐19 pandemic, there was very little mention of this in responses. However, change fatigue was very evident and consideration of our workforce is needed as local and international findings indicate a high proportion of emergency nurses intend to leave emergency nursing within 5 years, which will exacerbate pre‐existing shortages (Cornish, Klim, and Kelly 2021).

One of the key components of our implementation strategy was to focus on the everyday processes that could be enhanced by local clinical leaders (facilitators). The strategies were intended for consistent application to ensure implementation fidelity through the appointment of HIRAID facilitators or ‘implementers’. Most facilitators were part of the existing nursing workforce at the cluster and in a position to support the change as part of their roles, such as the cluster Clinical Nurse Educators and Clinical Nurse Consultants.

This study has generated evidence for implementation in the emergency context that considers emergency nurse capability, motivation and opportunity, addressing the gap in evidence in this field that has resulted in many large trials failing to effectively change emergency nursing practice (Middleton et al. 2019; McInnes et al. 2020). Since the conduct of this study, the HIRAID intervention has been implemented at all 30 study sites (Curtis et al. 2023). To determine if we generated a reliable implementation strategy to embed HIRAID into policy and practice for upscaling and system‐wide change we need to analyse factors influencing implementation, ecological validity and usability. We will measure implementation fidelity, that is the extent to which an intervention is delivered per the intended implementation plan (McGee et al. 2018; Slaughter, Hill, and Snelgrove‐Clarke 2015). This will be achieved through analysis of the additional formal feedback measures instigated during the implementation for research purposes: audits, weekly implementation team meetings, implementation logs and surveys of nursing staff who actively work in the emergency department.

Should the HIRAID trial improve patient and health service outcomes, and the implementation strategy be effective, reliable and relevant, we plan to upscale our findings using the methods presented in the manuscript with our research partners who have considerable influence on Australian health policy, emergency care delivery and nursing practice, such as the Australian Commission for Safety and Quality in Health Care, College of Emergency Nursing Australasia, Australian College of Nursing and Australian Government Office of the Chief Nurse and Midwife (Curtis et al. 2023).

### 6.1 Methodological Considerations and Limitations

The sites included rural, regional and metropolitan sites from different health jurisdictions and thus these findings may not be generalisable, and local context should be considered in the implementation of HIRAID or similar interventions in emergency care settings. Informal conversations and meetings with site leaders and stakeholders were held as part of site engagement prior to data collection, thus not discussed in this paper.

## 7 Conclusion

Implementation of interventions in emergency care settings is notoriously difficult. Respondents acknowledged there was a need for change in emergency nursing care delivery and were willing to learn and adopt something new, if it would improve patient care, if the intervention were evidence‐based and if they were adequately supported. A systematic process guided by the behaviour change wheel resulted in the generation of a multifaceted implementation strategy containing 22 behaviour change techniques to implement HIRAID across rural, regional and metropolitan emergency departments. Implementation fidelity, reach and impact of our strategy now requires evaluation.

## 8 Clinical Resources

HIRAID website https://www.sydney.edu.au/medicine‐health/our‐research/research‐centres/project‐hiraid.html. HIRAID https://aci.health.nsw.gov.au/networks/eci/research/current‐research‐and‐quality‐activities/hiraid. Behaviour Change Wheel https://www.behaviourchangewheel.com/. Video library for implementation https://www.youtube.com/@implementationsciencevideo6769.

## Author Contributions

K.C., J.C., M.M., R.Z.S. and M.F. contributed to the conceptualisation. K.C., J.C., S.K., R.Z.S. and M.F. contributed to the design and methodology of the work. K.C., J.C., R.Z.S. and M.F. contributed to validation. K.C., J.C., S.K., R.Z.S., C.A., J.A.H. and M.F. contributed to the funding acquisition. K.C., S.K. and M.K.L. contributed to analysis. B.K. contributed to resources and implementation mapping. K.C., B.K., M.M., R.Z.S. and C.A. contributed to project administration. K.C., S.K., R.Z.S., C.A. and M.F. contributed to original drafting. All authors contributed to writing, revisions and read and approved the final manuscript.

## Ethics Statement

Ethics approval was obtained for New South Wales sites through the Greater Western Human Research Ethics Committee (2020/ETH02164), and for Victoria through the Royal Brisbane & Woman's Hospital Human Research Ethics Committee (2021/QRBW/80026).

## Consent

Staff member indicated their consent using the online form. Responses were anonymous and the research team were unable to re‐identify participants from their responses.

## Conflicts of Interest

The authors have no competing interests to declare. The authors declare that they have no known competing financial interests or personal relationships that could have appeared to influence the work reported in this paper.

### Peer Review

The peer review history for this article is available at https://www.webofscience.com/api/gateway/wos/peer‐review/10.1111/jan.16461.

## Supporting information


Appendix S1.

Appendix S2.

Appendix S3.

## Data Availability

The datasets generated and/or analysed during the current study are not publicly available due to ethical constrictions but are available from the corresponding author on reasonable request to the ethics committee. The data contain potentially sensitive information in a large dataset. Data cannot be shared publicly because of ethical considerations.
