# Supplementary material for: Data‐Driven Implementation Strategy to Optimise Clinician Behaviour Change at Scale in Complex Clinical Environments: A Multicentre Emergency Care Study
Source: J Adv Nurs. 2024 Sep 15;81(5):2701–21. doi: 10.1111/jan.16461 (PMC11967304; doi:10.1111/jan.16461)
Supplement: Supplementary file 1 — Appendix S1. [file JAN-81-2701-s002.docx]

**Supplementary file 1.**

**Participant characteristics**

- Current Position
- Years worked as nurse
- Years in ED
- Highest qual
- ED areas worked most
- Facility

**Behavioural diagnostics**

- What are the best ways for you to learn about how to do something new?
  - Face to face education
  - Opportunity to ask questions
  - Feedback from my manger or educator about how I was performing)
  - Online learning
  - Hands on practice
  - Other
- Do you think using the same structured approach to assess patients would be beneficial in your ED? (Yes/No/Unsure)
  - If no, why?
    - There is no benefit to a standardised process
    - A single method will not suit all situation in the ED
    - Current practice is adequate
    - We all work differently, a single method doesn't suit all nurses
    - Other
- Are you willing to learn and adopt something new? (Yes/No/Unsure)
- Please indicate if you agree or disagree with the following statements about why you would or wouldn’t want to learn and use HIRAID
  - Not enough time to change the way of working
  - I don't have the headspace to learn something new
  - Too hard to remember anything new
  - It will not change the way I care for my patient
  - Unsupported by management
  - Nothing will change
  - I don't want to learn something new
  - The way we do things is fine, no need to change anything
  - I want to do what is best for patient care
  - I am worried I won't know what to do
  - I don't understand what HIRAID is
  - I am worried no one will help me with questions when I try and use it
- Please provide any additional reasons not provided above related to why you WOULD BE willing to learn something new
- Please provide any additional reasons not provided above related to why you WOULD NOT be willing to learn something new
- If you had to use HIRAID in your ED, is there anything that would help ensure it is implemented properly so it works?
  - Support in the clinical environment to adjust (people around to answer questions I might have
  - Visual prompts to remind me (posters)
  - Opportunity to ask questions
  - Feedback from my manager or educator about how I was performing
  - A policy
  - Knowing that there are consequences if I don't change
  - Knowing that the change is being monitored
  - Knowing that it will improve care for my patients
  - Opportunity to be part of the process of change
  - Other
