# Supplementary material for: Data‐Driven Implementation Strategy to Optimise Clinician Behaviour Change at Scale in Complex Clinical Environments: A Multicentre Emergency Care Study
Source: J Adv Nurs. 2024 Sep 15;81(5):2701–21. doi: 10.1111/jan.16461 (PMC11967304; doi:10.1111/jan.16461)
Supplement: Supplementary file 2 — Appendix S2. [file JAN-81-2701-s001.docx]

**Supplementary file 2: Figures and Tables**

# Figure 1. Mapping of identified TDF domains to Intervention functions (adapted from Michie (2014))


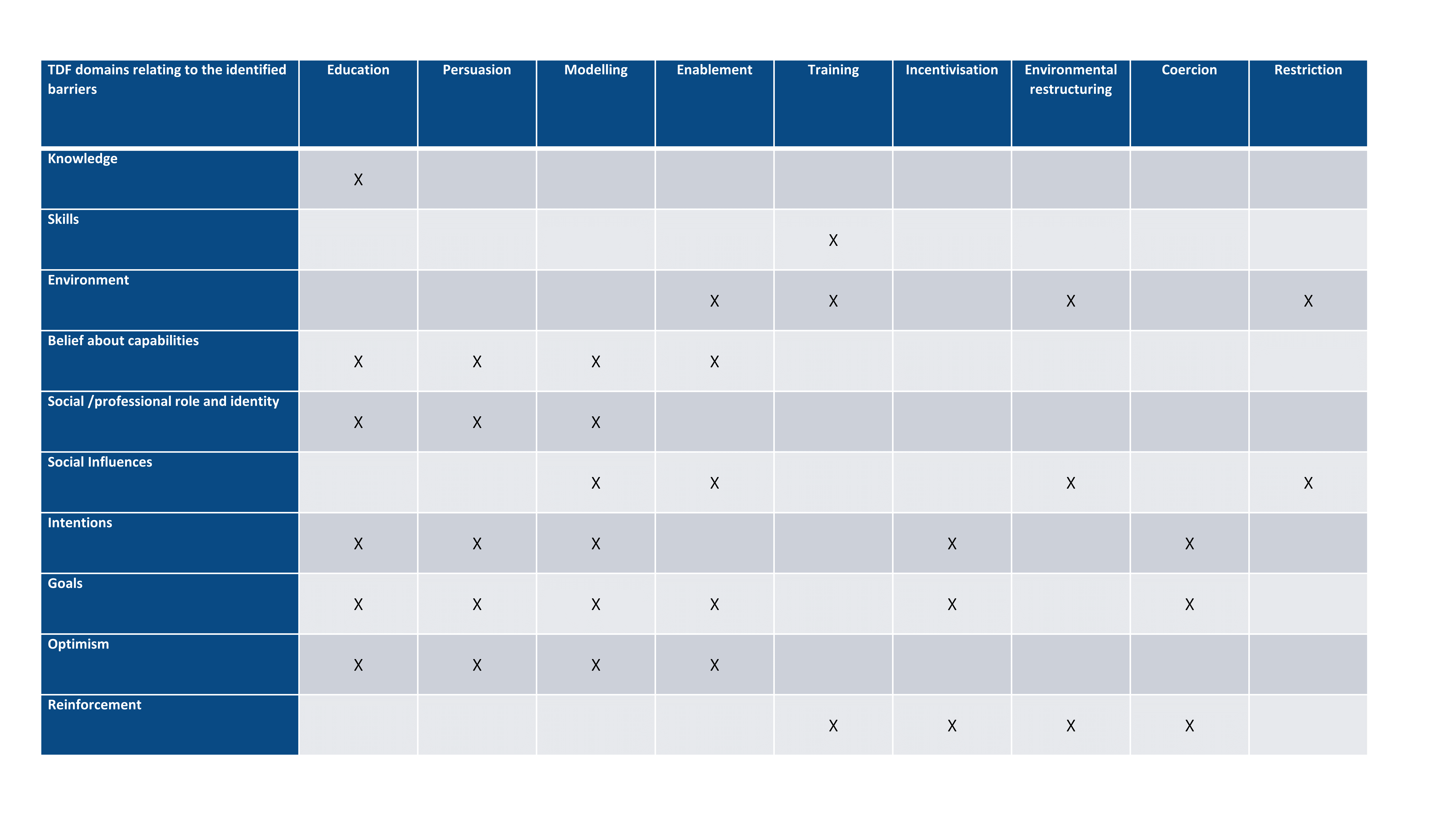


# Supplementary Table 1: Themes and sub-themes from free text survey responses regarding HIRAID® implementation with indication if preliminary barrier (B) or enabler (E)

| Theme (Barrier (B) or Enabler (E)) | Number of clusters (references) |
| --- | --- |
| **Implementation of HIRAID® will benefit patient care and nursing practice (E)** | 4 (111) |
| *A standardised approach will be beneficial (E)* | 3 (16) |
| *Will improve efficiency (E)* | 1 (7) |
| *HIRAID® improves patient care (E)* | 4 (48) |
| *Will support and / or enhance nurse practice (E)* | 4 (35) |
| *It is time for change (E)* | *3 (5)* |
| **Workload issues impacting implementation (B)** | 4 (88) |
| *Clinical time constraints (B)* | 4 (40) |
| *Intervention increases workload (B)* | 3 (18) |
| *Lack of staff resources (B)* | 3 (10) |
| *Need physical resources (B)* | 8 (4) |
| *Will increase documentation time away from patient (B)* | 2 (12) |
| ***Organisational and staff barriers will hinder change (B)*** | 4 (54) |
| *Change is difficult (B)* | *4 (25)* |
| *Poor workplace culture and burnout (B)* | *4 (5)* |
| *There is a lack of support for staff and education (B)* | 4(24) |
| **Nurses are motivated to learn and implement evidence (E)** | 4 (62) |
| *Nurses are motivated for evidence-based practice (E)* | 4 (40) |
| *Nurses are very willing to learn (E)* | 4 (22) |
| **Uncertainty of HIRAID® utility and concept (B)** | 4 (56) |
| *Familiar with but unsure of HIRAID® application (B)* | 3 (10) |
| *HIRAID® is principally documentation (B)* | 4(22) |
| *Uncertain HIRAID® will be of benefit (B)* | 4(24) |
| **A single tool restricts reasoning and is inflexible to context (B)** | 4 (27) |
| *Negative impact to clinical reasoning and judgement (B)* | 3(12) |
| *Standardised framework or approach does not suit ED (B)* | 3 (15) |

# Supplementary Table 2. Integration of quantitative and qualitative Barriers (B) and enablers (E) to HIRAID® implementation against TDF domains

| Barriers (B) / Enablers (E) | Supportive quantitative evidence | Supportive qualitative evidence | TDF domain |
| --- | --- | --- | --- |
| Lack of knowledge of HIRAID® (B) | “I don't understand what HIRAID® is”   - Overall 39.3% agree   Clusters 1,2,4  A structured approach is beneficial (78.1%) | Uncertainty of HIRAID® utility and concept (n=56)  *“I have no idea what HIRAID® is”* (Cluster4, P65)  *“There has been substantial change over the past years – even during covid – we are all mentally exhausted. The pressures have been relentless” (Cluster4, P99)* | Knowledge |
| High workload and perceptions of increased workload from HIRAID®(B) | “There is not enough time to change the way of working”   - Overall 35.9% agree Cluster 3 highest 45.3% | Workload issues impacting implementation (n=88)  *“…the main hurdle introducing HIRAID® to a Regional ED is TIME to document, which requires adequate staffing Levels and I can't see that ever changing as we are currently at breaking point already with the workload /expectations placed on us.” (Cluster3, P16)*  *“having already used HIRAID® I find it time consuming taking away time that can be used for other things such as procedures and patient care. I have seen other nurses spend 20-30 minutes on completing HIRAID® information, a lot of it not relevant to the presentation.” (Cluster1, P32)* | Environmental context and resources  Optimism |
| HIRAID® will not change anything (B) | “Nothing will change”   - Overall 30.7% agree   “It will not change the way I care for my patient”   - Overall 25.6% agree - Cluster3 highest 37.8% | Organisational and staff barriers will hinder change (n=54)  “*I would like to know the reasons WHY the change is being made and how it will improve things. If this information is not provided I will be less inclined to try and learn a new system” (Cluster3, P132)* | Belief about consequences  Social /professional role and identity |
| HIRAID® not suitable for workplace (B) | “The way we do things is fine no need to change anything”   - 13.3% agree | A single tool restricts reasoning is inflexible to context (n=27) | Environmental context and resources |
| Unsure of what to do (B) | “I am worried I won't know what to do”   - 23.2% overall agree - Barrier at 2 of 4 clusters   Metro clusters (Cluster3 and Cluster4) higher than regional (p=0.01) | Uncertainty of HIRAID® utility and concept (n=56) | Skills |
| Lack of support to implement or receive education (B) | “Unsupported by management”   - Overall 21.8% - Cluster2 + Cluster3 >20%   “I am worried no one will help me with questions when I try and use it”   - 20.7% agree | Workload issues impacting implementation (n=88)  *“Being adequately supported in the implementation of new practices. As opposed to the current environment where we have grossly inadequate staffing, no permanent management team on site, no educator at the facility for over 12months and increasing lack of medical officers with expected scope of practice with no resources or remuneration.”* (Cluster1, P10)  Organisational and staff barriers will hinder change (n=64)  “*If it requires significant time to develop new skills to assess patients this will only contribute more to staff stress level - we literally do not have the time on the floor to start over again so if it isn't quick and easy then it will be hard - everyone needs to be on board including medical staff so if they arent that will be difficult”* (Cluster4, P116)  “*I wont be given enough time or practice to learn anything new before it is implemented and subsequent changes (eg like in EMR) get sent through on an generic email, no education etc ...... no budget is a feature of NSW Health 'implementation' strategies .....” (Cluster2, P42)* | Social Influences  Environment |
| Nurses willing to learn and adopt something new (E) | “Willing to learn and adopt something new”   - Overall 89.7% agree   *“*I don’t have the headspace to learn something new”   - 90.3% disagree   *“It’s too hard to remember anything new”*   - 94.5% disagree   *“I don't want to* learn *something new”*   - 97.2% disagree | Nurses are motived to learn and implement evidence (n=62)  “Excited about change and improvement of practice. I am excited to learn new things” (Cluster3, P4)  *“That it is a collaborative process and exact examples of the process for various presentations would be beneficial” (Cluster2, P100)* | Intentions |
| Recognition of need to change way of working (E) | “The way we do things is fine, no need to change anything”   - 86.7% disagree   “A structured approach is beneficial”   - 78.1% agree | Implementation of HIRAID® will benefit patient care and nursing practice (n=111)  *“The current system is very broken. Anything that might help to improve it is worth trying. There is far too much variability in care provided at present, it can be a game of knob lotto when it comes to the patient experience with nurses.” (Cluster4, P86)* | Social/professional role and identity |
| The intervention will aid nurses to do what is best for patient care (E) | “I want to do what is best for patient care”   - 94.3% agree   “Knowing that it will improve care for my patients”   - 81.1% agree   *“A structured approach is beneficial”*   - 78.1% agree | Implementation of HIRAID® will benefit patient care and nursing practice (n=111)  Nurses are motivated to learn and implement evidence (n=62)  *“Always wanting to improve & work within current policy & best practice” (Cluster2, P46)* | Goals  Motivation |

**Supplementary Table 3. Behavioural diagnostics electronic survey questions**

| Participant characteristics | Current Position  Years worked as nurse  Years in ED  Highest qual  ED areas worked most  Facility |
| --- | --- |
| Behavioural diagnostics  What are the best ways for you to learn about how to do something new? | Face to face education  Opportunity to ask questions  Feedback from my manger or educator about how I was performing  Online learning  Hands on practice  Other |
| Do you think using the same structured approach to assess patients would be beneficial in your ED? | (Yes/No/Unsure) |
| If no, why? | There is no benefit to a standardised process  A single method will not suit all situation in the ED  Current practice is adequate  We all work differently, a single method doesn't suit all nurses  Other |
| Are you willing to learn and adopt something new? | (Yes/No/Unsure) |
| Please indicate if you agree or disagree with the following statements about why you would or wouldn’t want to learn and use HIRAID | Not enough time to change the way of working  I don't have the headspace to learn something new  Too hard to remember anything new  It will not change the way I care for my patient  Unsupported by management  Nothing will change  I don't want to learn something new  The way we do things is fine, no need to change anything  I want to do what is best for patient care  I am worried I won't know what to do  I don't understand what HIRAID is  I am worried no one will help me with questions when I try and use it |
| Please provide any additional reasons not provided above related to why you WOULD BE willing to learn something new | Free text |
| Please provide any additional reasons not provided above related to why you WOULD NOT be willing to learn something new | Free text |
| If you had to use HIRAID in your ED, is there anything that would help ensure it is implemented properly so it works? | Free text |
